# Enuresis

**Published:** 1872-04

**Authors:** 


*Enuresis.*

Dr. Woodman, in a clinical lecture at the London Hospital, published in the *Medical Press*, recommends the following method of investigating cases:

1st. Inquire carefully into the duration and frequency of the incontinence, and into the previous history of the child and the family.

2nd. Carefully note the diathesis, and general bodily condition of the patient.

3rd. Examine the *urine* by test papers, by other tests, and by the microscope if necessary.

4th. Make a careful examination of your little patient, including the anus and rectum, amongst the pudenda, and if needs be, exploring the bladder also. I need not enjoin upon you all possible delicacy and tenderness in these examinations. You must explain the necessity to the parents—and you will often astonish them by evidences of neglect of cleanliness, or of the existence of sores of which they were ignorant.

5th and lastly. Suit your treatment to the case; removing or soothing all causes of irritation, and at the same time endeavoring to improve the general health, for it is quite true that delicate and tubercular children, or those weakened by long and severe illnesses, such as the exanthemata, suffer most. Above all things *do not countenance corporal punishment for this* bodily infirmity. It is as cruel as it is usually useless; and I am in the habit of saying, that the parents or nurses ought to be beaten, and not the little patients.

Dr. W. mentions the following *auxiliaries* to treatment, useful in a great number of cases.

There is first the warm-bath; a sitting-bath is all that is generally needed. It should not be *too* warm, indeed 90° or at all events 98° is quite warm enough. Next, I have found it of great service to forbid the little patients to drink anything after their tea (say at five or six o'clock), unless thirst be extremely pressing.

On the importance of *waking* them up once, twice, or more in the night to make water, I need not dwell. This is not so cruel as it seems; the child is soon asleep again in most cases. It is also well not to let them lie on the back. The trigone of the bladder is the most sensitive part, and besides, it is possible the posterior regions of the spinal cord get unduly congested. A reel (such as used for cotton), fixed in the middle of the back sometimes prevents this, and the child may be turned over on the side.

For local sores, the glycerine of tannin, aided by cleanliness, is very valuable. I wish it did not stain the sheets so much. As tonics, mineral acids with bark, and the perchloride of iron, are the best I know; and if sweetened, children seldom refuse to take them, which is a great advantage.

As a sedative, belladonna is, perhaps, often better than opium. You may give from one-eighth to one-fourth of a grain of the extract even to very young children, three or four times a day. This is the most successful empirical remedy I know, and in obscure cases always deserves a trial, increasing the dose within the limits of safety. Should the urine, however, be saccharine, ammoniacal, or phosphatic, opium must have the preference.

For ascarides you will use local injections. Salt and water enemata are as good as anything else. In epileptic cases, cod-liver oil (if the fits are tubercular in origin) will perhaps deserve to be considered as more than an auxiliary.

In a communication to the *British Medical Journal*, Dr. Savage states that to a healthy girl, æt. 6½, who, from infancy, had been addicted to bed wetting, he ordered the lower part of her spine to be sponged daily, and with the most satisfactory result.—*The Doctor*.

